# Cathodal Transcranial Direct Current Stimulation Improves Focal Hand Dystonia in Musicians: A Two-Case Study

**DOI:** 10.3389/fnins.2017.00508

**Published:** 2017-09-12

**Authors:** Sara Marceglia, Simona Mrakic-Sposta, Manuela Fumagalli, Roberta Ferrucci, Francesca Mameli, Maurizio Vergari, Sergio Barbieri, Alberto Priori

**Affiliations:** ^1^Fondazione IRCCS Ca' Granda Ospedale Maggiore Policlinico Milan, Italy; ^2^Dipartimento di Ingegneria e Architettura, Università degli Studi di Trieste Trieste, Italy; ^3^Istituto di Bioimmagini e di Fisiologia Molecolare, Consiglio Nazionale delle Ricerche Segrate, Italy; ^4^“Aldo Ravelli” Center for Neurotechnology and Experimental Brain Therapeutics, University of Milan Milan, Italy; ^5^Department of Health Sciences, University of Milan and ASST Santi Paolo e Carlo Milan, Italy

**Keywords:** cathodal transcranial direct current stimulation, neuromodulation, tDCS, focal hand dystonia, musician

## Abstract

Focal hand dystonia (FHD) in musicians is a movement disorder causing abnormal movements and irregularities in playing. Since weak electrical currents applied to the brain induce persistent excitability changes in humans, cathodal tDCS was proposed as a possible non-invasive approach for modulating cortical excitability in patients with FHD. However, the optimal targets and modalities have still to be determined. In this pilot study, we delivered cathodal (2 mA), anodal (2 mA) and sham tDCS over the motor areas bilaterally for 20 min daily for five consecutive days in two musicians with FHD. After cathodal tDCS, both patients reported a sensation of general wellness and improved symptoms of FHD. In conclusion, our pilot results suggest that cathodal tDCS delivered bilaterally over motor-premotor (M-PM) cortex for 5 consecutive days may be effective in improving symptoms in FHD.

## Introduction

Focal hand dystonia (FHD) in musicians is a movement disorder characterized by irregularities in playing due to involuntary muscular activation in both the hand and arms (Cho and Hallett, 2016; Stahl and Frucht, 2017). FHD generally occurs in people who have spent a long period of time performing repetitive skilled motor tasks (Cho and Hallett, 2016). Furthermore, FHD produces excessive co-contraction of agonists and antagonists of hand and forearm muscles resulting in a slow, stiff-appearing movement and causing pain (MacKinnon, 2002; Garraux et al., 2004). Being a network disorder that involves several brain areas, FHD has a complex pathophysiology including several general abnormalities as the loss of inhibition, sensory dysfunction, and abnormal plasticity (Cho and Hallett, 2016). Functional neuroimaging studies showed alterations in the topography and increased activation of somatosensory and motor cortices (Zeuner and Molloy, 2008; Hinkley et al., 2009). Even though the causes of this disabling condition remain unclear, maladaptive plasticity has been proposed as driver for FHD in musicians (Konczak and Abbruzzese, 2013).

Despite several new therapeutic strategies proposed, botulinum toxin injection is the preferred therapy for FHD, even though some results suggest that it does not effectively improve symptoms (Hallett et al., 2009; Lungu and Ahmad, 2016). External shock waves therapy (Trompetto et al., 2009), prolonged immobilization of the affected arm (Priori et al., 2001), thalamic deep brain stimulation (Cho et al., 2009), repetitive Transcranial Magnetic Stimulation (rTMS) (Edwards et al., 2008), and transcranial Direct Current Stimulation (tDCS) (Cho and Hallett, 2016) are among the alternatives explored.

In particular, tDCS is a non-invasive technique that induces prolonged changes in brain excitability and influences motor and cognitive performances (Nitsche and Paulus, 2000; Priori, 2003; Ardolino et al., 2005; Priori et al., 2008; Brunoni et al., 2013; Zhao et al., 2017). In the past 10 years, tDCS has been proposed as adjunctive treatment for several neurological and neuropsychiatric conditions (Fregni et al., 2006; Ferrucci et al., 2008a,b; Monti et al., 2008; Mrakic-Sposta et al., 2008; Cogiamanian et al., 2009; Lefaucheur et al., 2017).

Cathodal tDCS was applied in FHD, but the results were controversial, mainly for the heterogeneity of the stimulation protocols tested (Buttkus et al., 2010, 2011; Benninger et al., 2011; Furuya et al., 2014), in terms of electrode montage, tDCS duration and intensity, as well as the number of treatments administered. Also, tDCS was either performed when the patient was at rest, or while the patient was trained (Cho and Hallett, 2016). Biparietal tDCS (two electrodes with different polarities on the head) applied over days during neurorehabilitation could improve therapy effectiveness in FHD (Furuya and Altenmüller, 2015; Rosset-Llobet et al., 2015) and bilateral tDCS (two electrodes with the same polarity on the head) is thought to improve symptoms in subjects showing bi-manual impairments (Pixa et al., 2017). Evidence of increased excitability or loss of inhibition at multiple levels including premotor and motor cortex, somatosensory cortex and cerebellum (Beck et al., 2008; Brighina et al., 2009; Delnooz et al., 2012) support the hypothesis that cathodal tDCS could improve symptoms by reducing excitability, even though the optimal protocol is still to be determined (Cho and Hallett, 2016).

In this pilot study, we applied bilateral anodal, cathodal, and sham tDCS over the motor—premotor cortices for 5 consecutive days in two musicians with FHD, to (1) test whether cathodal tDCS was superior to anodal and sham tDCS in controlling FHD symptoms in musicians, and (2) preliminarily assess the use of a 5-days protocol in terms of safety and efficacy.

## Methods

### Patients

After signing their informed consent, two musicians with FHD were recruited for tDCS treatment application. The study was approved by the local Ethic Committee and it was in agreement with the principles stated in the Declaration of Helsinki. All the assessments were performed by an experienced neurologist and the diagnosis was made according to the recommendations found in the literature (Rosset-Llobet et al., 2009).

#### Patient 1

The first patient was a right-handed 38 years-old man, in whom FHD symptoms manifested first at the age of 18. He began playing piano when he was 11 years-old. At present, he teaches in a secondary school and plays as concert pianist.

Musician's FHD was diagnosed at the age of 22, but the disease progressively exacerbated. In the past time, the therapeutic intervention with Transcutaneous Electrical Nerve Stimulator (TENS) did not improve symptoms. Anatomic Magnetic Resonance Imaging (MRI) brain scan was normal and electroencephalogram (EEG) did not show any sign of epileptic seizures. At the time, focal dystonia interested the middle finger of the right hand and the left hand.

Presently, despite the drug treatment (anticholinergics: 12 mg/day, carbidopa/levodopa: 500 mg/day), dystonic tremor is present at rest and when the right hand is tired or weak (Lee et al., 2015).

#### Patient 2

The second patient was a right-handed, 44-years-old man. He began playing the accordion at the age of 11. At present, he teaches in a secondary school and plays saxophone, accordion, and clarinet.

First symptoms (cramps) appeared on the right hand at the age of 30. The patient was treated with botulinum toxin injections in hand and forearm muscles without any improvement.

At present, FHD symptoms were predominantly on the right hand, particularly the fifth finger with a subjective complaint extending to the wrist and the distal forearm. The patient complained initial symptoms to the left hand, too. The patient is not currently taking any pharmacological treatment. Anatomic MRI was normal and EEG did not show any sign of epileptic seizures. Electroneurography (ENG) showed normal motor and sensory conduction parameters; electromyography (EMG) showed a reduced disynaptic and presynaptic inhibition of H reflex in the right flexor carpi radialis (0 ms = 100%; 20 ms = 86%), compatible with a diagnosis of FHD.

### Clinical assessment at baseline

Musician's FHD was evaluated at baseline (see Table 1) using the following rating scales: the *Symptom Severity Scale (SSS)* that consists of 10 questions that evaluate direct and indirect disease manifestations; the *Functional Status Scale (FSS)*, a 12-item disability scale that comprises an assessment of performances of daily activities possibly affected by FHD or hand weakness. The SSS and FSS, which were originally designed for carpal tunnel syndrome, were adapted to FHD in order to investigate its manifestations.

**Table 1:** Clinical Examination at baseline in both patients.

**CLINICAL EXAMINATION T0 (Baseline Evaluation)**		
**Scale**	**Patient 1**	**Patient 2**
FSS	11/36	2/36
TC	3	2
SSS	28/43	24/43
FMS	2	3
MMPI-2	Hy 74 (cut-off ≤ 65; *z* = 2.6)	Normal
CBA	IP-F (*z* = −1.73)	Normal
	IP-1 (*z* = −1.9)	
	MOCQ/R1 (*z* = 2.23)	

*FSS, Functional Status Scale; TC, Tubiana and Chamagen Scale; SSS, Symptom Severity Scale; FMS, Fahn Marsden Scale; MMPI-2, Minnesota Multiphasic Personality Inventory Scales; [Hy, Hysteri]; CBA, Cognitive Behavioral Assessment [IP-F = Fear; IP-1 = Fear: Calamity; MOCQ/R = Obsessions and compulsing:checking]*.

The *Fahn Marsden Scale (FMS)* (Fahn, 1989) was used to quantify generalized or focal dystonia in nine body areas, including eyes, mouth, speech and swallowing, neck, trunk, and right and left arm and leg. The *Tubiana and Chamagne (TC)* (Tubiana and Chamagne, 1983) scale is a classification of severity of focal dystonia in musicians, and it was used to monitor the evolution of the treatment. The scale comprises four stages of severity of dystonia.

Psychological assessment was executed using the *Minnesota Multiphasic Personality Inventory Scales (MMPI-2)* (Butcher et al., 2001) and the *Cognitive Behavioural Assessment 2.0 (CBA-2.0)* (Sanavio and Vidotto, 1996). The MMPI-2 is a well-known and widely used psychological test consisting of 567 true-false items. It traditionally yields scores on four validity scales and 10 clinical scales, although numerous other scales may be scored. For this study, the clinical, select content (Anxiety, Depression, Negative Treatment Indicators) and supplementary (Ego Strength) scales were considered. The CBA 2.0 battery includes a series of questionnaires that investigate broad issues of potential clinical interest and identify areas of dysfunction in the current life of the subjects. Subjective mood, wellness and pain at the hands were evaluated using five 100 mm *Visual Analog Scales (VAS)* (happy/unhappy; wellness/unease; left hand pain/no left hand pain; right hand pain/no right hand pain, tired/no tired).

### Performance assessment

The effects of tDCS on FHD were evaluated through the following tasks administered before and after each tDCS treatment:

#### Copy of an archimedes spiral

Patients were asked to copy a spiral template printed in black on a paper with their dominant arm. The template was 132.31 cm long. The quantitative value was defined through the length of the drawn Archimedean spiral: L = pi^*^N^*^R with N = R/t; where: *t* = spiral's step, *N* = number of rpm, *R* = max radius of spiral. We compared the spiral template length to the length of the drawn spiral. The presence of tremor was qualitatively evaluated by four independent judges blinded to the stimulation polarity on a 0–3 scale (0 = absence of tremor, 1 = slight tremor, 2 = medium tremor, 3 = important tremor).

#### Follow with a pen the edge of the spiral

Patients were asked to follow the spiral line with the pen maintaining a constant distance of 2 mm from the printed line. The task was evaluated by four judges blinded to the stimulation polarity on a 0–3 scale about the presence of tremor (0 = absence of tremor; 1 = slight tremor, 2 = medium tremor, 3 = important tremor).

#### Copy of sample pictures

Patients had to copy different figures (cube, pyramid, and more complex figures) and a 10 cm line. Four blind judges evaluated the presence of constructive apraxia on a 0–3 scale (0 = perfect copy; 1 = very similar copy, 2 = incomplete copy, 3 = very incomplete copy) and the presence of tremor (0–3 scale with 0 = absence of tremor, 1 = slight tremor, 2 = medium tremor, 3 = important tremor).

#### Copy of a list of words

Eight lists of 33 words were created using the Lists for Writing. Each list was composed by words (verbs, adjectives, concrete and abstract nouns, all of them both regular and irregular) and non-words. Four blind judges evaluated the quality of writing on a 0–1 scale (0 = good writing; 1 = bad writing symptom of a weak and tired hand), and the presence of tremor (0–3 scale with 0 = absence of tremor, 1 = slight tremor, 2 = medium tremor, 3 = important tremor). The % difference from pre-treatment evaluation was considered for the analysis.

#### Execution of a musical scale and exercises with the instrument

Patients executed a musical scale and several technical exercises with their instrument. More specifically, for the piano, the patient was asked to play a two-octave C-major scale, right and left hand, 10 sequences; for the clarinet, the patient was asked to play a two-octave C-major scale.

Adverse events were collected throughout the whole session.

### Experimental protocol

**Figure 1:**
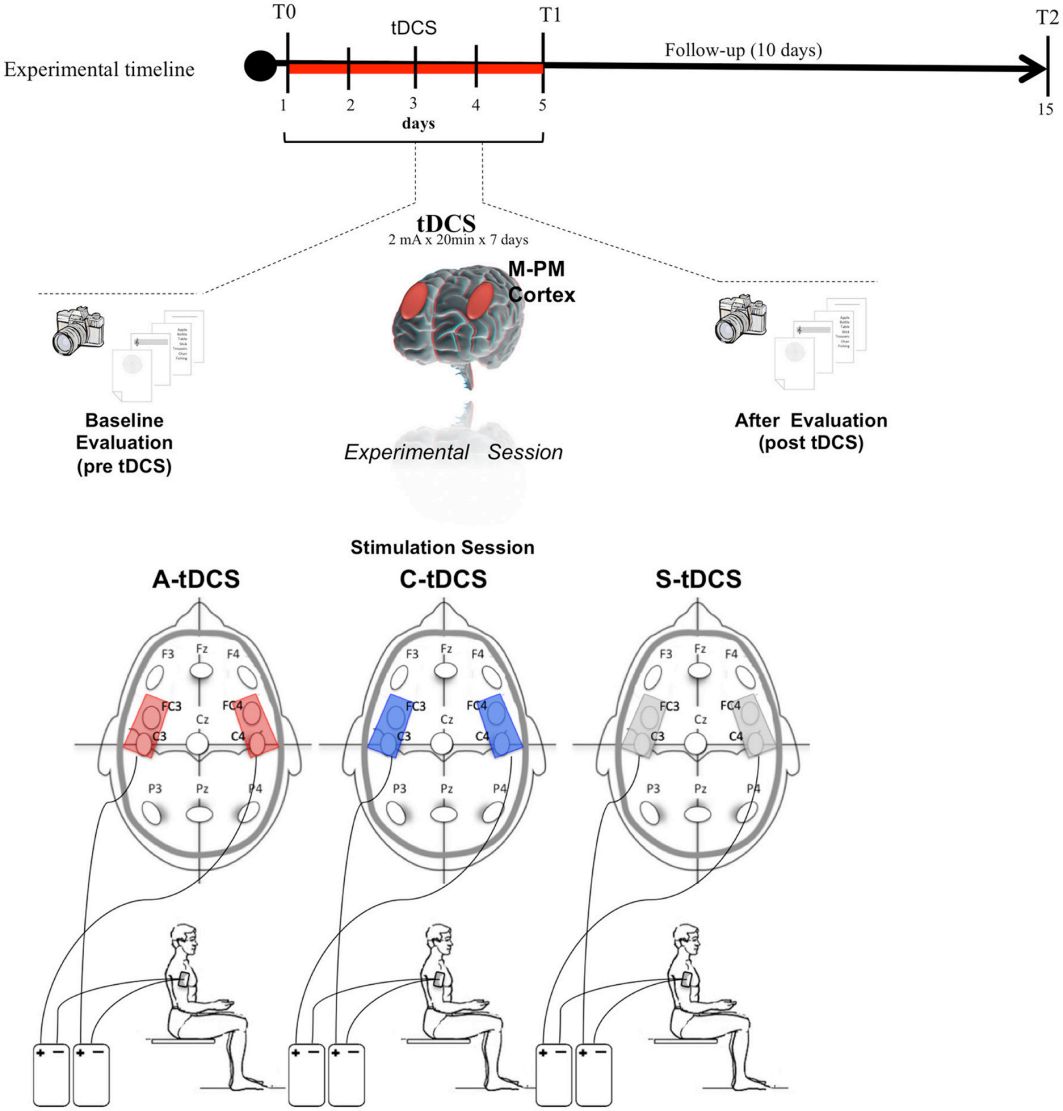
Transcranial Direct Current Stimulation (tDCS) experimental protocol. tDCS was applied bilaterally over the scalp on the motor-premotor cortex (M-PM) for 5 consecutive days. Patients were assessed in the first (T0) and the last day (T1) and after 10 days from ended tDCS (T2).

This was a double-blind experiment in which both the patients and the judges were blind to the type of stimulation delivered. Both patients underwent three 5-days sessions, one for sham, one for cathodal, and one for anodal tDCS, in random order (Figure 1). Three washout weeks elapsed between each session (anodal, cathodal, sham). After a baseline clinical assessment at the beginning of the 5-day session, each day included (1) pre-tDCS performance evaluation; (2) bilateral tDCS at 2 mA intensity per side over the motor/premotor areas (M-PM) of the cerebral cortex (above C3/C4, FC3/FC4 according to the international 10–20 electrode placement system) for 20 min; and (3) post-tDCS performance evaluation. At the end of the 5-days session, FHD symptoms were re-assessed through the SSS, the TC, and the FSS (Figure 1).

Follow-up examinations, including both symptom and performance assessments were conducted at 1 (T1) and 10 (T2) days after the end of each 5-days session.

### tDCS

tDCS was delivered to the scalp with two “Eldith DC Stimulator” (Neuroconn GmbH, Germany), each connected to a pair of thick (0.3 mm) rounded saline-soaked sponge electrodes, one (active electrode) placed over the scalp and the other (reference electrode) over the right deltoid muscle. Cathodal and anodal tDCS (C-tDCS and A-tDCS) polarity referred to the two electrodes over the scalp. The wide electrode surface (scalp electrodes 48 cm^2^; deltoid electrode 64 cm^2^) avoided the possible harmful effects of high current density. For sham tDCS (S-tDCS), electrodes were placed as for real stimulation but the stimulator was turned off after 10 s. Hence, the patients felt an initial itching sensation similar to that induced at the beginning of real tDCS but received no stimulation.

To guarantee safety we applied, to each stimulation site, current at a density of 0.0416 mA/cm^2^ and delivered a total charge of 0.049 C/cm^2^. These intensities are far below the threshold for tissue damage (Nitsche et al., 2003a; Liebetanz et al., 2009; Lefaucheur et al., 2017).

### Data analysis

Considering the low number of subjects reported, we performed only descriptive statistics.

Percentage changes, defined as [(after tDCS-before tDCS)/before tDCS], were used for the analysis to assess tDCS effects. When applicable, the percentage changes of variables evaluated by the independent judges were averaged, to obtain the trend over the entire 5-days session. Data are reported as [mean ± *SD*].

## Results

The patients did not report any adverse effects during stimulation sessions and were not able to distinguish active (anodal or cathodal) and sham stimulation.

### Copy of an archimedes spiral

**Figure 2:**
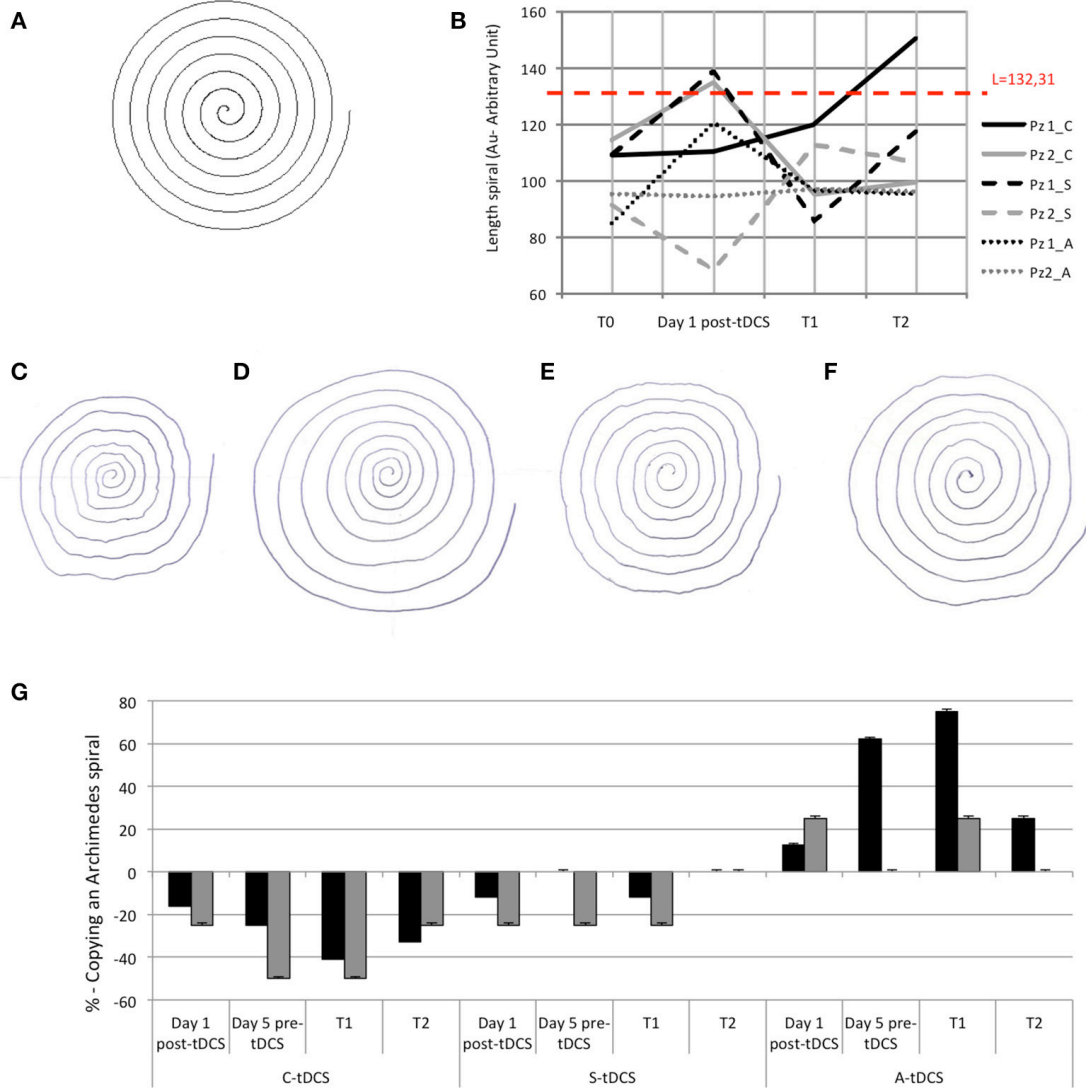
**(A)** Copy of an Archimedes spiral task: template of Archimedean Spiral; **(B)** The lines show quantitative evaluation in both patients at T0, at day 1 after tDCS, at T1, and at T2. The red dashed line is the length of the sample spiral (132.31 mm), black lines refer to patient 1 and gray lines to patient 2. The full is C-tDCS, the dashed line is A-tDCS and the dotted line s S-tDCS session; **(C)** Copy of Archimedean Spiral the first day after C-tDCS treatment in patient 1; **(D)** Copy of Archimedean Spiral at T1 of the C-tDCS session in patient 1; **(E)** Copy of Archimedean Spiral at the first day after A-tDCS treatment in patient 1; **(F)** Copy of Archimedean Spiral at T1 of the A-tDCS session in patient 1. **(G)** The histogram shows tremor in qualitative analysis in the percentage differences after tDCS in both patients. 0% is baseline (pre-tDCS—T0).

Quantitative evaluation showed that whereas S-tDCS and A-tDCS left patients' performance unchanged, after C-tDCS both patients could draw a complete *Copy of an Archimedes spiral* (template length 100%, Figure 2A). The time elapsing before each patient could draw a complete spiral differed in the two patients: patient 1 drew a complete spiral 1 day after the entire 5-day C-tDCS session, whereas patient 2 achieved a complete spiral immediately after the first C-tDCS application (day 1) in the post-C-tDCS assessment. Conversely, when sham and anodal sessions ended, both patients could draw a spiral that was 20–30% shorter than the printed one. At T2, the beneficial effect of C-tDCS ended (Figure 2B). Qualitative analysis showed that C-tDCS reduced tremor, whereas A-tDCS and S-tDCS did not (Figures 2C–G).

### Follow with a pen the edge of the spiral and copy of a sample pictures

**Figure 3:**
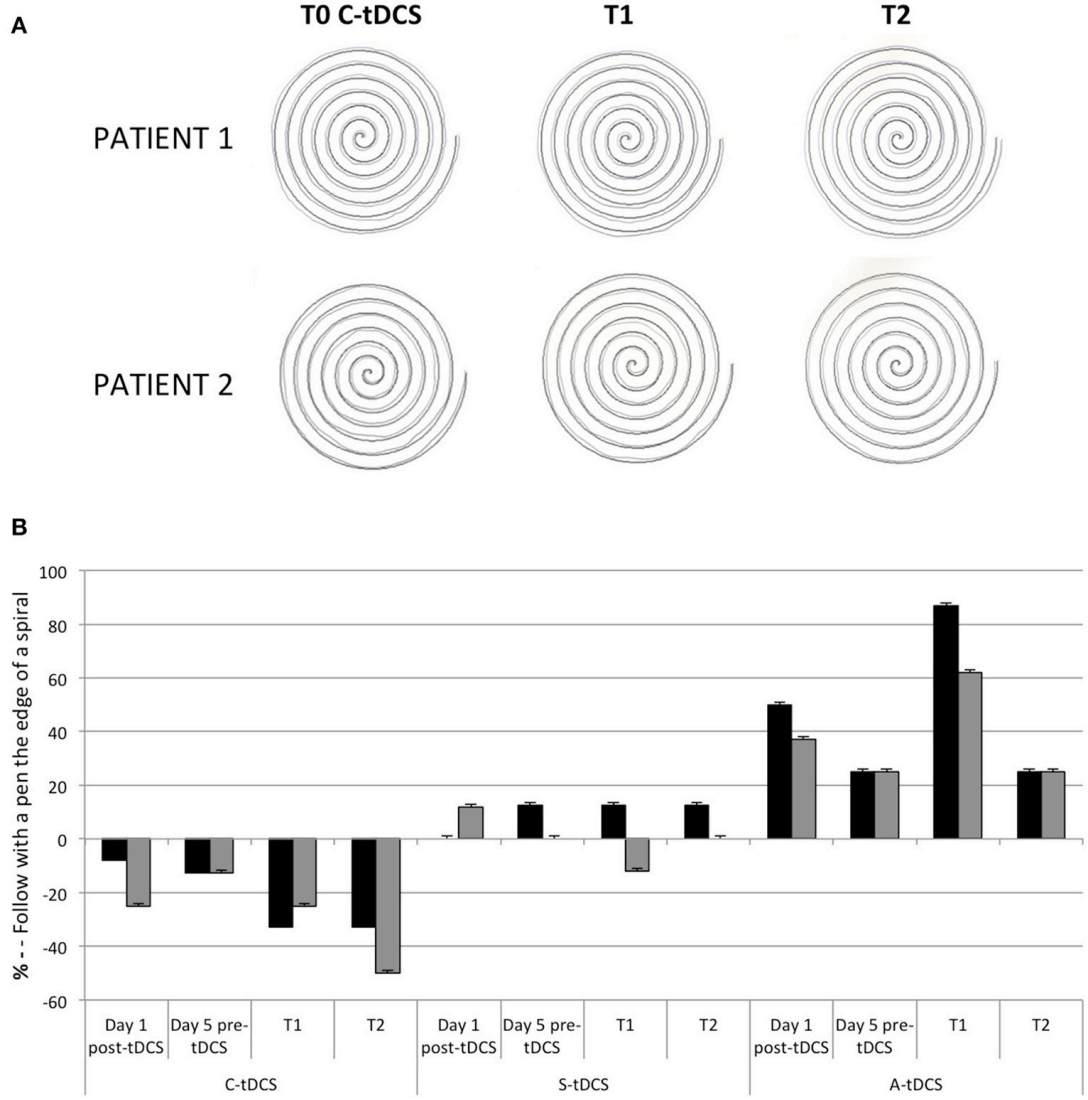
**(A)** Follow with a pen the edge of the spiral at pre (T0) and post C-tDCS (T1 and T2) in both patients. **(B)** The histogram showed the qualitative analysis in the percentage differences after tDCS in both patients. 0% is baseline (pre-tDCS—T0).

In the *Follow the edge of a spiral with a pen* task both patients improved at T1 of the C-tDCS session and worsened after A-tDCS (Figures 3A,B).

**Figure 4:**
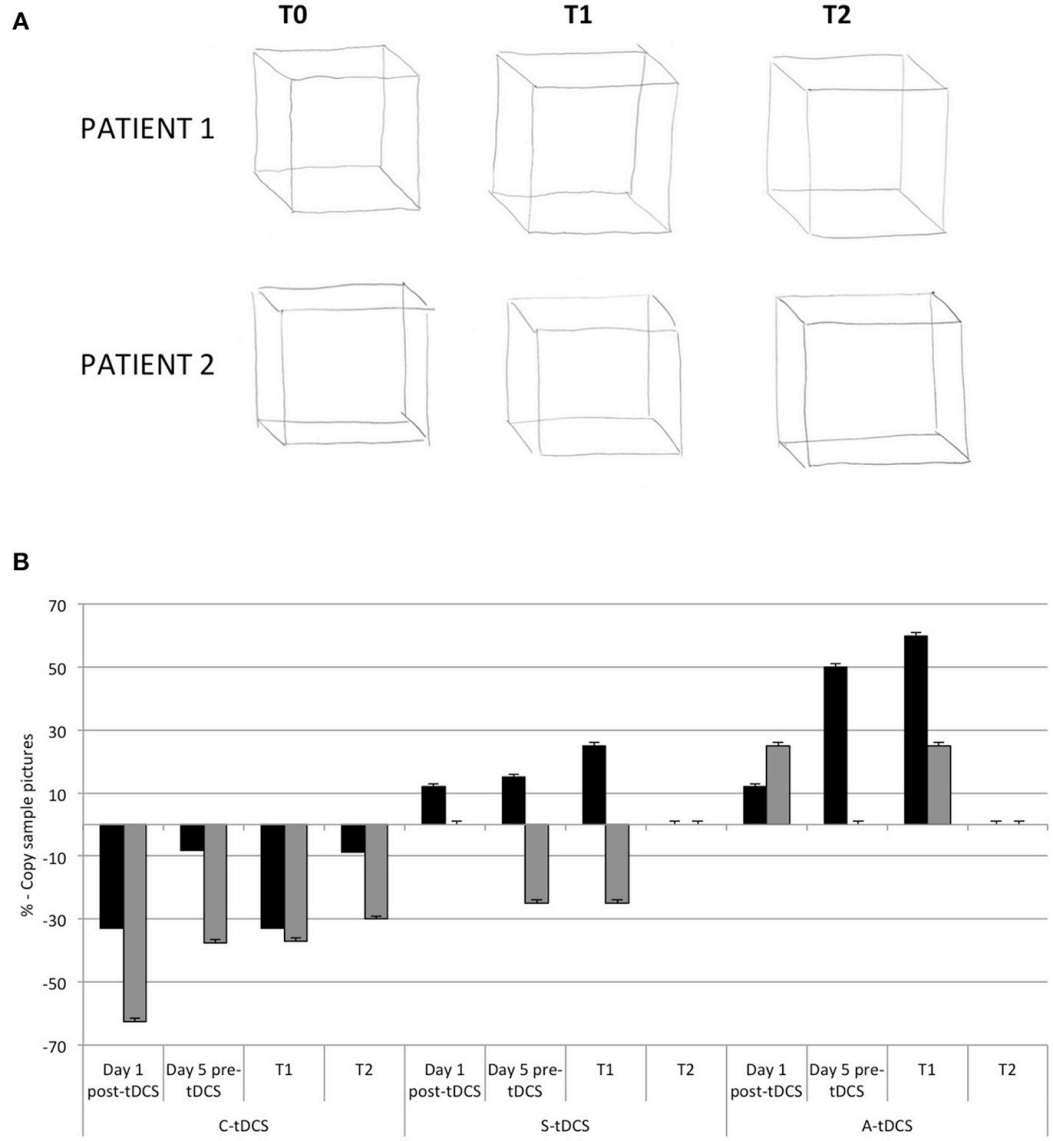
**(A)** The tremor graft pre (T0), and post (T1 and T2) C-tDCS in both patients. **(B)** The histogram showed qualitative analysis in the percentage differences of tremor after tDCS in both patients. Zero percent is baseline (pre-tDCS—T0).

In both patients, tremor decreased (patient 1: −33%; patient 2: −62%) while patients were “*copying sample pictures*” immediately after a single C-tDCS session. At T2, the effect of C-tDCS persisted (% change at T2 C-tDCS, Patient 1: −9%; patient 2: −30%; Figures 4A,B).

### Copy of a list of words

Either way, before and after tDCS (anodal, cathodal, and sham), the quality of writing was good in both patients, with no presence of bad writing due to a weak or tired hand and tremor.

### FHD symptoms and playing

FSS, TC, and SSS values improved in both patients after C-tDCS. At T1, FSS decreased by 9% in patient 1 and 100% in patient 2, TC decreased by 33% in patient 1 and 100% in patient 2, SSS decreased by 3% in patient 1 and 46% in patient 2. After A-tDCS, only some values improved: FSS decreased in patient 1 by 35 and 100% in patient 2, TC did not change in patient 1 and 2, SSS decreased by 13% in patient 1 and 5% in patient 2 (Table 2).

**Table 2:** Clinical assessment of both patients before and after 5-days tDCS session.

**CLINICAL ASSESSMENT**												
	**T0 (Before tDCS)**						**T1 (5 days after tDCS)**					
**Scale**	**Patient 1**			**Patient 2**			**Patient 1**			**Patient 2**		
	**C-tDCS**	**S-tDCS**	**A-tDCS**	**C-tDCS**	**S-tDCS**	**A-tDCS**	**C-tDCS**	**S-tDCS**	**A-tDCS**	**C-tDCS**	**S-tDCS**	**A-tDCS**
FSS	11/36	12/36	17/36	2/36	2/36	2/36	10/36	11/36	11/36	0/36	0/36	0/36
TC	3	3	3	2	2	2	4	3	3	4	2	3
SSS	28/43	28/43	29/43	24/43	17/43	20/43	27/43	29/43	25/43	13/43	18/43	19/43

*FSS, Functional Status Scale; TC, Tubiana and Chamagen Scale; SSS, Symptom Severity Scale*.

**Figure 5:**
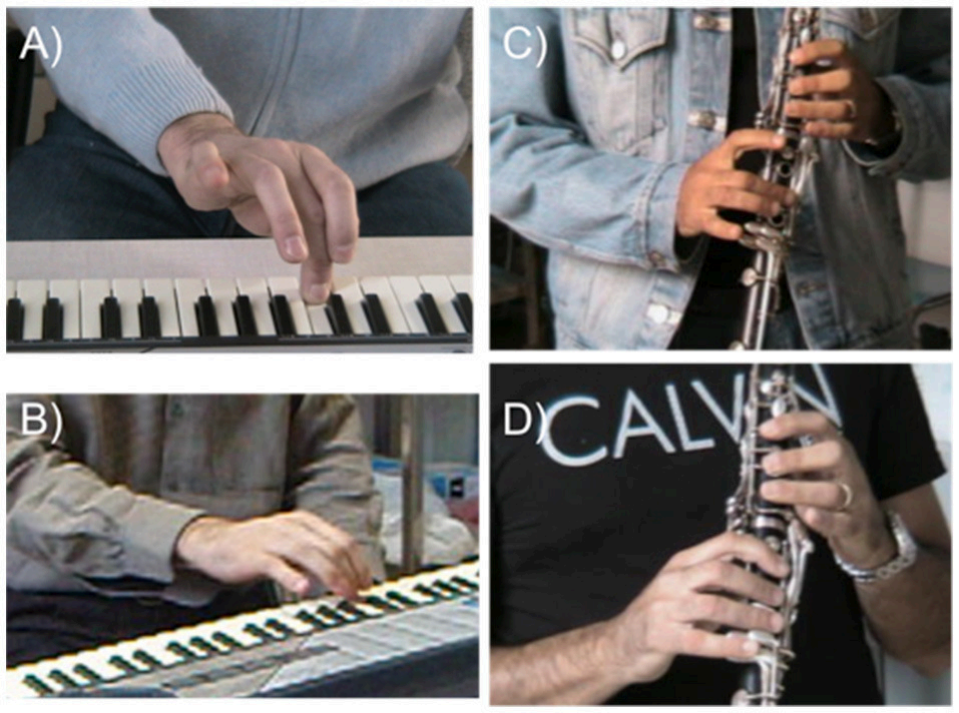
Typical patterns of dystonia posture in a pianist **(A)** and clarinet **(C)** at baseline (T0). In **(B,D)** the patients after 5-days of C-tDCS (T1).

Patient 1 reported a subjective improvement and decrease of pain at the right hand only after C-tDCS. He also reported subjective improvement of prono-supination movement in the left hand (Figures 5A,B).

Patient 2 reported a subjective improvement after cathodal tDCS: the right hand was more toned, he had a better control of the 5th finger, fewer shots, and less hand/art fatigue (Figures 5C,D).

### VAS

Both patients reported a sensation of general wellness, of happiness, of reduction of hand pain and an improvement of rest particularly after C-tDCS (at T1). In details, for health/wellness: patient 1, 21 vs. 32 vs. 19% (S-C-A-tDCS, respectively); patient 2, 0 vs. 4 vs. 0%; happy/unhappy: patient 1, 20 vs. 55 vs. 43%; patient 2, 0 vs. 8 vs. 2%; pain/no pain right hand patient 1, 13 vs. 37 vs. 0%; patient 2, 0 vs. 0 vs. 0%; pain/no pain left hand patient 1, 2 vs. 68 vs. 3%; patient 2, 0 vs. 0 vs. 0%; tired/not tired patient 1, 18 vs. 42 vs. 24%; patient 2, 0 vs. 5 vs. 7%).

## Discussion

Musician's FHD is a difficult disease, with little therapeutic options while bringing to early termination of their professional life. We showed that cathodal tDCS delivered bilaterally for 5 consecutive days is feasible and safe, and that it transiently improves motor performances, subjective perception of pain and fatigue, and subjective perception of playing in two musicians with FHD.

Our findings, while proposing a new stimulation protocol, are in line with the conclusions of the recent review by Cho and Hallet who reported the potential therapeutic use of non-invasive brain stimulation to treat FHD (Cho and Hallett, 2016). Considering the current state-of-the-art, there are few studies investigating the effects of cathodal tDCS in FHD, but none of them applied a 5-days bilateral (two electrodes with the same polarity on the head) M1 tDCS protocol nor it found improvements lasting for 10 days. More specifically, of the seven tDCS studies mentioned in the review (Rosset-Llobet et al., 2009; Buttkus et al., 2010, 2011; Benninger et al., 2011; Furuya et al., 2014; Sadnicka et al., 2014), four have applied electrodes on M1 but only one reported positive effects (Furuya et al., 2014). Unlike the others, the authors combined cathodal tDCS on the affected M1 with anodal tDCS on the unaffected M1 in pianists, finding that rhythmic accuracy of sequential finger movements improved and was retained 4 days after intervention.

Following the Furuya S and colleagues' suggestion of working on the two sides of the head contemporarily, we applied tDCS bilaterally (two electrodes with the same polarity on the two head sides) over the M-PM cortex in two musicians with FHD and bilateral symptoms, sharing the idea of a possible advantage over mono-lateral stimulation. The M-PM cortex was chosen as tDCS target because it is believed to encode the motor programs responsible for skilled finger movement (Karni et al., 1995; Gentner et al., 2010). In addition, bilateral tDCS was shown to improve symptoms occurring on both sides of the body (Pixa et al., 2017), similarly to our two patients, who had both hands affected by symptoms (at least in an initial phase). Then, considering the positive results of applying tDCS for one week (5 days) in other hyperkinetic disorders (Mrakic-Sposta et al., 2008), and considering the boosting effects of tDCS applied over days during neurorehabilitation (Furuya and Altenmüller, 2015; Rosset-Llobet et al., 2015), we chose the 5-days protocol.

After 5 consecutive days of cathodal tDCS, both our patients experienced consistent improvements in tremor, slight improvement of finger postures during playing, and diminished pain in the hands and arms, without experiencing any side effect. Moreover, our patients, who were blinded to the treatment received, reported a positive subjective impression of how C-tDCS affected their pain, tiredness, mood, and wellness. Conversely, in line with the study by Quartarone et al. (2005), where anodal tDCS was shown to up regulate brain excitability in patients with writer's cramp, A-tDCS worsened the symptoms in our patients. Collectively, our results suggest that cathodal tDCS treatment might help to produce steady, more accurate arm movements, but not to stabilize abnormal fine finger movements. Because cathodal tDCS reduces brain excitability (Nitsche et al., 2003b), bilateral cathodal tDCS delivered over the motor areas could have down regulated excitability in the underlying brain areas by recovering the inadequate motor cortical inhibition responsible for excessive excitation and near synchronous co-contractions of agonists and antagonists (Nitsche et al., 2003b; Ardolino et al., 2005; Byl, 2007). Our application of bi-hemispheric C-tDCS seemed to help recovering patient's bilateral symptoms and overall condition. We can hence hypothesize that, in presence of bilateral symptoms, tDCS should be delivered bilaterally (two electrodes with the same polarity on the two head sides) whereas, in presence of unilateral symptoms (Furuya et al., 2014), tDCS preferable application may be bipolar (two different polarities on the head). Moreover, the 5-days protocol resulted to be effective in providing beneficial effects at least for 2 weeks, confirming that over-days tDCS sessions may be more effective than single-shot tDCS sessions in promoting brain plasticity, especially if combined with neurorehabilitation therapy (Rosset-Llobet et al., 2015).

Finally, our experiment differs from all the others reported in the literature for both the electrode montage (bilateral tDCS) and the stimulation protocol (5 consecutive days). This could partly explain why our results are more consistent than those obtained by other groups (Cho and Hallett, 2016).

However, this was a double-subject studies that allows observing individual responses, but not generalizing its results to the whole population of FHD musicians, as inherent limitation of the experimental design. Although, no definitive conclusions can be derived from 2 subjects, our results should be interpreted in the context of the novel target of stimulation (bilateral M-PM for patients with initial bilateral symptoms) and stimulation protocol (5 consecutive days) that may be considered as new factors in future trials. Also, we haven't assessed any biomarker to better understand the potential pathophysiological mechanisms of tDCS that should be included in further studies.

In conclusion, a 5-days treatment with cathodal tDCS could be a safe and low-cost effective adjuvant in the therapy of involuntary flexion or extension of hand and limbs and in hand pain and the bilateral electrode montage over PM-M areas could favorably impact FHD in musicians with bilateral symptoms.

## Ethics statement

The study was carried out in accordance with the recommendations of the Ethical Committee of the Fondazione IRCCS Ca'Granda Ospedale Maggiore Policlinico with written informed consent from all subjects. All subjects gave written informed consent in accordance with the Declaration of Helsinki.

## Author contributions

SM and SMS designed the study, conducted the experiment, analyzed data, and drafted the manuscript and figures. MF, RF, and FM designed and administered the cognitive tasks and reviewed the manuscript. MV administered tDCS and reviewed the manuscript. SB evaluated the patients, provided clinical advice and scales, and reviewed the manuscript. AP ideated the study, coordinated the research protocol, and finalized the manuscript.

### Conflict of interest statement

SM, SMS, RF, MF, MV, and AP are co-founders of Newronika srl, a spin-off company of the Fondazione IRCCS Ca' Granda Ospedale Maggiore Policlinico and the University of Milan, Milan, Italy. The other authors declare that the research was conducted in the absence of any commercial or financial relationships that could be construed as a potential conflict of interest.
